# Optic nerve sheath ultrasonography in the setting of laparoscopic surgery

**DOI:** 10.1016/j.bjane.2026.844789

**Published:** 2026-07-01

**Authors:** Varun Suresh, Rohan Magoon, Nitin Choudhary

**Affiliations:** a Sheikh Jaber Al Ahmad Al Sabah Hospital, Department of Anesthesia and Intensive Care, Kuwait, Arabian Gulf; b Atal Bihari Vajpayee Institute of Medical Sciences (ABVIMS) and Dr. Ram Manohar Lohia Hospital, Baba Kharak Singh Marg, Department of Anaesthesia and Cardiac Anaesthesia, New Delhi, India; c All India Institute of Medical Sciences (AIIMS), Department of Anaesthesiology, Pain Medicine and Critical Care, New Delhi, India

*Dear Editor*,

The recent research on variations in Optic Nerve Sheath Diameter (ONSD) assessed non-invasively by Ultrasonography (USG) amongst patients undergoing laparoscopic prostatectomy merits the attention of readers.^1^ Application of non-invasively assessed ONSD variations in neurologic and non-neurologic scenarios is a rapidly evolving domain of knowledge.^2^ Given that intraoperative infusion of intravenous mannitol for patients with non-pathologic transient variations in intracranial pressure adds novelty to the research by Barreto et al, a few caveats in this research need further elaboration.^1^

To begin with, intra- or inter-observer variability and the imaging experience of the anesthesiologist can present an important limiting factor when sub-millimetric variations in USG-assessed ONSD are extrapolated to significant study outcomes. Herein, we note that the index authors assigned senior anesthesiologists with more than five years USG hands-on experience for intraoperative measurements and therefore, suggest a more tenable alternative.^1^ The GE HealthCare Logiq® USG systems are equipped with software to capture screenshots of ‘frozen’ images and store it for future analysis. Offline ‘blinded’ ONSD measurements can be randomly performed on such stored images outside the operating room by observers not directly involved in the patient management. We believe that this minor technical change in approach can render the ONSD measurements more methodologically robust with reduced observer-related variability.^3^

Further, a predominantly elder group of patients undergoing prostatic surgery in the index research attracts close attention.^1^ Ageing-related cerebral cortical atrophy can result in physiologically widened Cerebrospinal Fluid (CSF) spaces among this subgroup. These changes are compounded by astrocytic degeneration and septations in the CSF space that surround the optic nerve. Altogether this contributes to a physiologically wide CSF space on USG image which warrants special consideration while ONSD measurements are undertaken herein.^3^ Therefore, we assume that factors that affect plasticity of ONS need careful narration while results of ONSD measurements are elaborated in elderly.^1^^,^^3^

For explicit understanding and to substantiate this research context; we conducted a literature search on the point-of-care clinical utility of USG for ONSD estimation, to capture transient fluctuations in ICP in an elective setting as in the Barreto et al. study i.e., capnoperitoneum due to laparoscopy.^1^ Using MeSH search terms “Optic nerve sheath diameter” [AND] “Laparoscopy” in PubMed, we found 63 results which were downloaded as a Microsoft Excel spreadsheet. Citations metrics of the top 10 articles in this dataset were retrieved using iCite® tool of the National Institute of Health and is depicted in Table 1. The majority of top cited articles focused on ONSD measurements during laparoscopic/robotic prostatic surgery with consistent research interest in gynecologic and pelvic surgery. It is evident here that the application of ONSD as a noninvasive technique to estimate intraoperative variations in ICP, in completely non-neurologic scenarios, is attaining widespread readership attention and applause.^1^^,^^2^

**Table 1:** Citation counts of the top 10 articles in the field of optic nerve sheath diameter assessment during a Laparoscopic surgery setting.

Sl No	Year of publication	Title of the article	Journal Name	Total Citations^a^
1	2014	Increase in intracranial pressure during carbon dioxide pneumoperitoneum with steep trendelenburg positioning proven by ultrasonographic measurement of optic nerve sheath diameter	Journal of Endourology	72
2	2017	Changes in intraocular pressure and optic nerve sheath diameter in patients undergoing robotic-assisted laparoscopic prostatectomy in steep 45° Trendelenburg position	BMC Anesthesiology	46
3	2015	Detection of elevated intracranial pressure in robot-assisted laparoscopic radical prostatectomy using ultrasonography of optic nerve sheath diameter	Journal of Neurosurgical Anesthesiology	45
4	2019	Ultrasound optic nerve sheath diameter evaluation in patients undergoing robot-assisted laparoscopic pelvic surgery	Journal of Robotic Surgery	34
5	2014	Optic nerve sheath diameter remains constant during robot assisted laparoscopic radical prostatectomy	PLoS One	32
6	2019	Effects of pneumoperitoneum and steep Trendelenburg position on cerebral hemodynamics during robotic-assisted laparoscopic radical prostatectomy: A randomized controlled study	Medicine (Baltimore)	29
7	2018	Ultrasonographic optic nerve sheath diameter for predicting elevated intracranial pressure during laparoscopic surgery: a systematic review and meta-analysis	Surgical Endoscopy	23
8	2018	Effect of Pneumoperitoneum and Patient Positioning on Intracranial Pressures during Laparoscopy: A Prospective Comparative Study	Journal of Minimally Invasive Gynecology	23
9	2021	Comparison of different end-tidal carbon dioxide levels in preventing postoperative nausea and vomiting in gynaecological patients undergoing laparoscopic surgery	Journal of Obstetrics and Gynaecology	20
10	2018	Propofol attenuates the increase of sonographic optic nerve sheath diameter during robot-assisted laparoscopic prostatectomy: a randomized clinical trial	BMC Anesthesiology	19^b^

a Total citation here are inclusive only to PubMed data analyzed on 04 February 2026 (Google scholar citation counts may vary).

b Article titled “Elevation in optic nerve sheath diameter due to the pneumoperitoneum and Trendelenburg is associated to postoperative nausea, vomiting and headache in patients undergoing laparoscopic hysterectomy” published in Minerva Anestesiologica (2020) also received 19 citations, till date (as on 04 February 2026).

At the same time, there exists another pertinent technical intricacy with point-of-care ONSD measurements, that needs to be elucidated. B-scan ultrasound technique for ONSD measurements are prone to “Blooming effect” artifacts due to a lack of standard sensitivity setting in the ultrasound machine.^4^ Such effects are general technical limitations of bedside ONSD assessment that future research needs to address.^1^^,^^2^^,^^4^ The A-scan ultrasound features as an alternative imaging method wherein high reflective spikes can be visualized at the interface between the arachnoid membrane and the CSF; yet the logistic nuance of having a much larger module of ultrasonography inside a laparoscopic operating room cannot be forgotten.^4^^,^^5^

The research by Barreto et al is praiseworthy considering the novelty in methodology wherein they attempt pharmacologic ICP reduction with IV Mannitol among patients undergoing laparoscopy; with standardized peak inspiratory airway pressures and end-tidal carbon-dioxide levels throughout all four timeframes of their measurements.^1^ Nevertheless, the extent to which imaging technicalities can affect the ONSD measurements among a predominant geriatric cohort of patients, needs simultaneous emphasis. We suggest adherence to expert consensus on the bedside-imaging of ONSD as a ready reckoner to future research in the subject.^5^

## Data availability statement

No new data were generated or analysed in this article. Data sharing is not applicable to this article.

## IEC approval

IEC approval is NOT required.

## AI assistance disclosure

The authors declare that no generative artificial intelligence or AI-assisted technologies were used in the preparation of this manuscript.

## Funding

Not applicable.

## Conflicts of interest

The authors declare no conflicts of interest.
